# A hybrid algorithm for clinical decision support in precision medicine based on machine learning

**DOI:** 10.1186/s12859-022-05116-9

**Published:** 2023-01-03

**Authors:** Zicheng Zhang, Xinyue Lin, Shanshan Wu

**Affiliations:** 1 Nanjing University, Nanjing, China; 2 Shandong University, Jinan, China

**Keywords:** Information retrieval, BM25, BioBert, Abstract extraction, Machine learning

## Abstract

**Purpose:**

The objective of the manuscript is to propose a hybrid algorithm combining the improved BM25 algorithm, k-means clustering, and BioBert model to better determine biomedical articles utilizing the PubMed database so, the number of retrieved biomedical articles whose content contains much similar information regarding a query of a specific disease could grow larger.

**Design/methodology/approach:**

In the paper, a two-stage information retrieval method is proposed to conduct an improved Text-Rank algorithm. The first stage consists of employing the improved BM25 algorithm to assign scores to biomedical articles in the database and identify the 1000 publications with the highest scores. The second stage is composed of employing a method called a cluster-based abstract extraction to reduce the number of article abstracts to match the input constraints of the BioBert model, and then the BioBert-based document similarity matching method is utilized to obtain the most similar search outcomes between the document and the retrieved morphemes. To realize reproducibility, the written code is made available on https://github.com/zzc1991/TREC_Precision_Medicine_Track.

**Findings:**

The experimental study is conducted based on the data sets of TREC2017 and TREC2018 to train the proposed model and the data of TREC2019 is used as a validation set confirming the effectiveness and practicability of the proposed algorithm that would be implemented for clinical decision support in precision medicine with a generalizability feature.

**Originality/value:**

This research integrates multiple machine learning and text processing methods to devise a hybrid method applicable to domains of specific medical literature retrieval. The proposed algorithm provides a 3% increase of P@10 than that of the state-of-the-art algorithm in TREC 2019.

## Introduction

Precision medicine is a new medical paradigm that integrates modern scientific and technological means with conventional medical methods by detailing human bodily functions and the nature of diseases scientifically, thus optimizing systematically the principles and practices of human disease prevention and health care to eventually maximize both individual and social health benefits with more effective, safer, and more economical medical services [1, 2]. In precision medicine, diagnostic methods are appropriately selected for each patient to realize minimal iatrogenic damage, minimum medical costs, and optimal patient recovery [3, 4]. Besides, utilizing both genomic profiles and healthcare data sources of patients to a large extent leads to personalized treatments [5]. Hence, the clinical system adopting this new approach mainly pays attention to all types of useful information regarding genes, microbiomes, environmental conditions, family history, and lifestyles of patients to pick precise diagnoses and therapeutic alternatives that individually result in better treatments [6]. In other terms, precision medicine is considered a tool that could be used for several purposes such as predictive, preventive, personalized, and participatory healthcare service utilizing all available data sources such as genetics, omics, and patients’ history [7].

Precision medicine has been covering various areas ranging from drug discovery, design, and development, the analysis of drug sensitivity in pharmacology, and the construction of clinical decision support systems in health analytics to a better understanding of several diseases and their relationships with genes, family history, and other attributable factors in medicine [8–11].

With the advancement of medical technologies, the number of biomedical articles has grown exponentially. So, finding relevant articles matching the symptoms of a patient in massive article databases becomes increasingly difficult. For example, when just “precision medicine” is written in the search bar in the Science Direct database, the number of articles that are found is 229,126. Therefore, getting both useful and practical insights out of the immense collection requires to be implemented finely devised methods and approaches.

Information retrieval (IR) plays a significant role in precision medicine and refers to the process and technology to organize and access information according to the requirements of users. The main goal of information retrieval is to obtain the required information as accurately, quickly, and comprehensively as possible. Moreover, since data accumulation grows sharply, big data-based crunching and modeling have been gaining momentum, especially after 2008 [12]. Hence, more precise, and refined outcomes could be potentially reached by employing finely devised methods or algorithms.

Even though the BM25 algorithm is the first and most widely used algorithm to improve better algorithms in text ranking tasks, most BM25 algorithms only consider abstracts and do not consider the possible search morphemes and their co-occurrence relationships that could be found in chemicals, MeSH, and keywords. Zhang [13] proposed an improved BM25 algorithm that computes three scores for the vocabulary, co-word, and expanded word that leads to a composite retrieval function whose parameters are optimized by the cuckoo optimization algorithm that retrieved better search outcomes. The model was trained on the 2017 dataset. The results showed that the trained parameters produced improvements in the search results when both the 2018 and 2019 datasets are used, so this research provided a reference for parameter selection for the BM25 algorithm. Several of the available algorithms utilize the BM25 algorithm as the first step of a search algorithm and then employ a deep learning model to obtain more accurate matchings. Besides, it should be kept in mind that the effect of deep learning models is dependent on how well the models get trained. Therefore, similarity results could be highly affected by the results of the employed method in the first stage. Consequently, the improved BM25 used at the first stage provides advantages to attaining better search results in the proposed algorithm.

This manuscript will base on the improved BM25 approach to pick the highest scores of 1000 articles in PubMed and conduct a clustering algorithm to split into N different clusters to reach the minimum input requirement of the pre-trained model on the data set called BioBERT to generate better text ranking results by using search terms of diseases, genes, and individual traits. Therefore, similarity-matching results will be attained based on finally running the BioBERT model that is employed also as a pre-training model and calculates the similarity between the article abstract/title and the retrieval morpheme as a score. Due to the limitation of the input vector length of the BERT model which is restricted to using 512 tokens (words or characters) in an article abstract, negative samples for the training data set are generated to improve the training effect.

The motivation of the research is to propose a hybrid algorithm consisting of a two-stage information retrieval method based on the improved BM25 algorithm, k-means clustering, and BioBert model to better determine the most relevant biomedical articles to specific diseases, genes, and individual traits.

The sections of the article are organized as follows: Section "Related work" presents the related works. Section “Method” describes the improved BM25 algorithm, and proposed the algorithm whose stages are called document similarity matching, and cluster-based abstract extraction. Section "The Proposed Method and its Implementation" describes the proposed method with a flow chart and its execution details including data structure, and negative training sample generation method. Section"Experimental results" describes the experimental comparison results of the proposed algorithm and the selected algorithm presented in Track 2020, as well as the data and parameters used by the proposed algorithm. Section "Summary and future work" concludes the research.

## Related work

### Preliminary

In this subsection, we will present a brief introductory development of text retrieval. The Boolean model constitutes the search model of the original information, which was used for information retrieval as early as 1957 and is a simple retrieval model based on the set theory and Boolean algebra whose basic idea is to represent the query of a user and a document by utilizing a set of words. Then, the similarity of the two sets is determined by using Boolean operations. Moreover, the Boolean model is a keyword-matching type of information retrieval, that is, documents containing the keywords in a query will be retrieved. However, there exists usually a low correlation between the retrieved results and the target. In some research fields, weighting the index terms has been shown to greatly improve the retrieval results, which has led to the development of vector models [14, 15].

BM25 and its modified versions, which are characterized by conventional probabilistic models employing the two-Poisson approximation of the term-frequency distribution, have been long effective tools in text ranking and the BM25 algorithm is generally used to compare the performance of the newly introduced models [16, 17]. Besides, typical vector models include the term frequency-inverse document frequency (TF-IDF) approach and the BM25 model have been widely studied based on this approach. As a result, the emergence of vector models has substantially increased the relevance of retrieved documents to the retrieval target and led to the concepts of document scoring and ranking [18–20].

With the advancement of machine learning algorithms in recent years, several ranking algorithms have been developed by aiming at better ranking the texts in the search of matching the query with the most relevant articles. Besides, when machine learning algorithms are implemented, more automatic processes are expected to attain better outcomes. Learning-to-rank methods are generally classified into three categories according to the training methods: pointwise, pairwise, and listwise [21–23]. In the pointwise method, each document in the training set is treated as a separate sample, which is essentially a single-document classification and regression problem. Some widely implemented pointwise algorithms include Prank [24], McRank [25], and RankProp [26]. In the pairwise method, document pairs with different labels for the same query in the training set are trained as one sample. Based on two documents with different labels, the ranking problem is finally transformed into a binary classification problem. Some broadly utilized algorithms include the rank boost algorithm [27] and the frank algorithm [28]. In the listwise method, the entire document sequence is taken as a sample, and the evaluation of the information retrieved is optimized by defining a loss function. Some widely conducted research includes ListNet [29], SVMMAP [30], and the ADA rank algorithm [31].

When machine learning algorithms are implemented, the pre-training process contributes to the success of these algorithms [32–34]. A pre-trained language representation approach, called BERT (A multilayer bidirectional transformer encoder stack), was proposed by [35] and the BERT’s performance was found to be better than the available ones in the literature. Park et al. [36] used a bidirectional encoder representation from transformers (BERT) classifier to train retrieved articles and word vectors to represent medical articles. The studies were ranked according to similarity scores between query semantic elements and the article. The results showed that the accuracy was greatly improved over existing algorithms. Pan et al. [19] combined patient health records with biomedical articles and used three methods to expand the phrases used in queries, and the experimental results showed that the proposed model yielded a promising average weighted accuracy, better stability, and applicability. Maciej et al. [37] investigated the effectiveness of a BERT-based ranking model on different platforms. The results verified the accuracy of the BERT model for precision medicine too. Bayesian networks into query expansion and probabilistic models to expand query semantic elements to increase query accuracy were introduced [9]. Two types of BERT models, BERT_BASE_ and BERT_LARGE_, are available [38]. Some articles covering various related modifications of BERT can be found in [39–42].

### BioBert model

With the implementation of the BioBERT model [43–46], Natural Language Processing tasks extract better relations and generate more accurate outcomes. Instead of pre-training on generic data sets, BioBert requires derived data sets to perform well. On the contrary, poor performances would be expected. The BioBERT model is used for various improvement purposes. For example, the identification of functional links between proteins has been recently conducted by fine-tuning weights from BioBERT [44]. Besides, several research manuscripts have reported better outcomes when the BioBERT model is implemented [47–50] in the literature.

## Method

### Baseline algorithm

Our baseline algorithm employs the improved BM25 algorithm previously proposed by the author. To ensure the integrity of the paper, The fundamental aspects of the improved BM25 algorithm are revisited [13].

First, we defined the abstract score,

1 $$AS(Q,d)=\sum_{i}^{n}IDF({q}_{i})\times \frac{{f}_{i}\times \left({k}_{1}+1\right)}{{f}_{i}+{k}_{1}\times \left(1-{b}_{1}+{b}_{1}\times \frac{dl}{avgdl}\right)}$$

where Inverse Document Frequency (IDF) is the search morpheme ${q}_{i}$, where ${k}_{1}$ and ${b}_{1}$ are the adjustment factors, which are usually set according to the experience of users, ${f}_{i}$ is the frequency of ${q}_{i}$ in $d$. IDF is defined as follows: IDF for a particular word can be obtained by dividing the total number of documents by the number of documents containing the searched word and then taking the logarithm of the quotient. $dl$ is the text length of document $d$, and $avgdl$ is the average text length of all documents.

We propose a wordlist to combine the chemical words, MeSH headings, and keywords of a retrieved document, and the scores are defined as follows:

2 $$WS\left( {Q,d} \right) = \mathop \sum \limits_{i}^{n} \frac{{tfw\left( {Q,d} \right) \times \left( {k_{2} + 1} \right)}}{{tfw\left( {Q,d} \right) + k_{2} \times \left( {1 - b_{2} + b_{2} \times \frac{dwl}{{avgdwl}}} \right)}}$$

where $tfw$ is the sum of the IDF values of each retrieved morpheme, and k_1_ and b_1_ are adjustment factors, which are usually set according to the experience of users. $dwl$ is the number of words in the wordlist of document d, and $avgdwl$ is the average number of words in the wordlist of all documents.

We also defined the co-word score, that is, the disease and gene in the search morpheme (including expansion words) co-occur in the abstract, and the wordlist is recorded as the co-occurrence score as follows:

3 $$\mathrm{CWS}\left(Q,d\right)=\sum_{i}^{n}{IDF}_{word}({g}_{i},d)$$

where ${IDF}_{word}({g}_{i},d)$ represents the score based on the expression gene ${g}_{i}$ for query $Q$, the summation is used since some tasks could contain genes.

To achieve the same level as the scores of the similarity method in the manuscript, we standardize the sum of the three scores, and the standardization method adopts the max–min method, as shown in Eq. (4):

4 $${x}_{norm}=\frac{x-\mathrm{min}(X)}{\mathrm{max}(X)-\mathrm{min}(X)}$$

where ${x}_{norm}$ represents the normalized value,$x$ represents the value before normalization, $\mathrm{min}(X)$ represents the minimum value of the sequence to be standardized, and $\mathrm{max}(X)$ represents the maximum value of the sequence to be standardized. In the algorithm, we also added query expansion to extend the mesh. The algorithm and its performance evaluation in detail can be found in [13].

### Document similarity matching

**Fig. 1:**
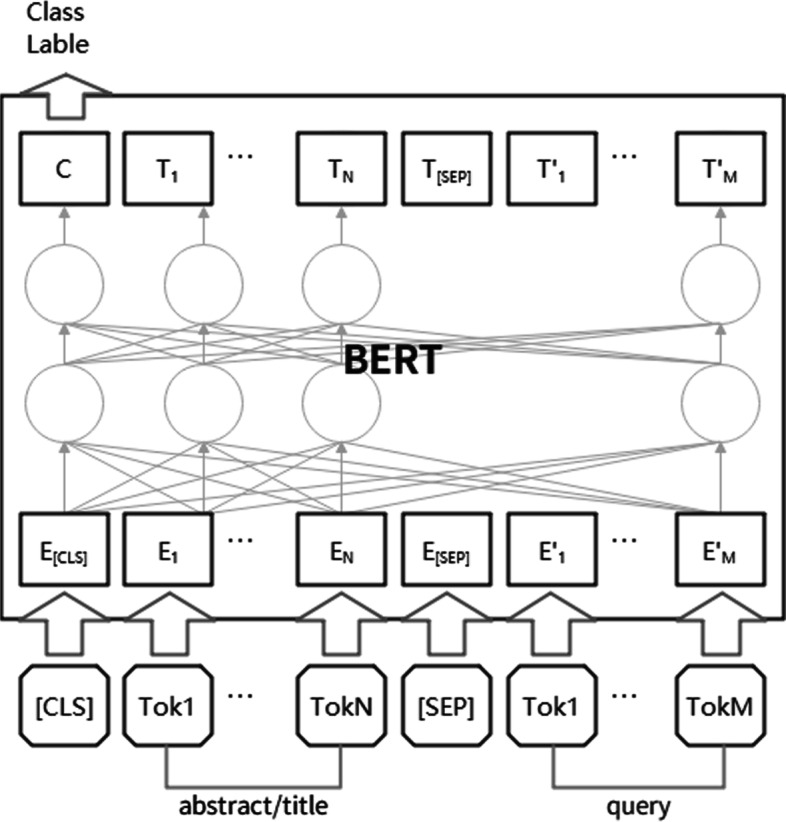
The classification task of sentence pairs in the BERT

Similarity matching between articles and retrieval tasks is an important step in the information retrieval process. In [24], Bidirectional Encoder Representation from Transformers (BERT) model is employed to train the abstracts/titles and query tasks. The model structure is shown in Fig. 1. [CLS], which is a special vector, is added to the top of the input before transferring and sending it to the BERT and [SEP], which is a special tag to separate sentences, is added as a separator between the abstract/title. Then, the output of the BERT model (the embedding of sentence pairs) is taken, and [CLS] is utilized to complete the similarity calculation task. The output sigmoid is computed to obtain the similarity between the abstract/title and the query, which is considered as the matching score between the input abstract/title and the query.

### Clustering-based abstract extraction

Because the BERT model is limited to 512 tokens (words or characters), the abstract needs to be further streamlined, and the key content needs to be extracted. An extractive abstract generation method is employed to preserve the writing style and the meaning of the original abstract to the highest extent. Then, the article adopts the clustering-based abstract extraction method, and the specific process is described as follows:

1. The BioBert pretraining model is utilized to generate a sentence vector for each sentence in the abstract to obtain a sentence-level vector representation, which is a 1 × 768 dimensional vector.

2. Sentences are clustered by using the K-means clustering to obtain N categories, where the number N is preassigned by the implementer.

3. A sentence closest to the center of the cluster is selected from the category until the overall length reaches 512 tokens (words or characters) to form a new abstract text.

## The proposed method and its implementation

### The proposed algorithm

**Fig. 2:**
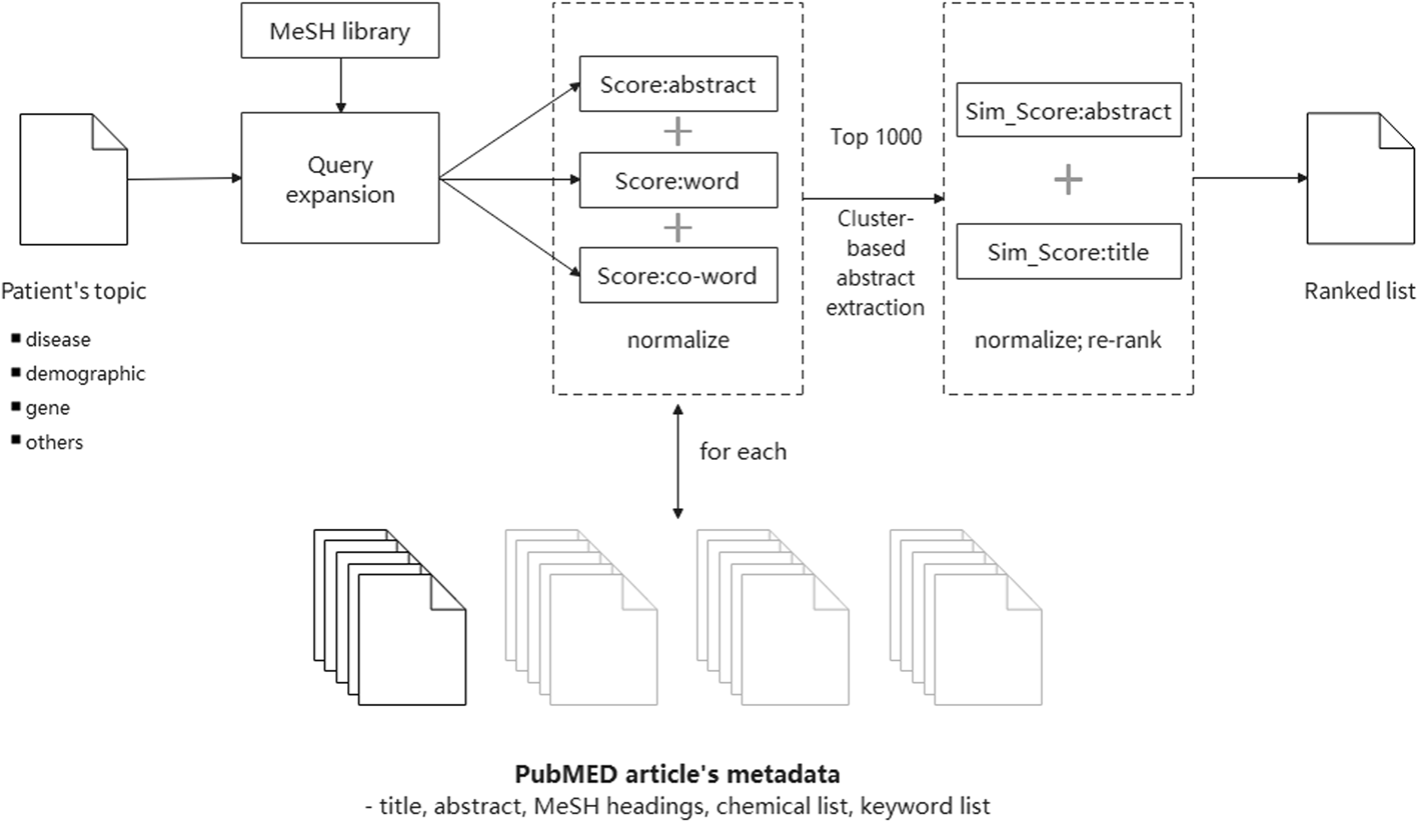
Algorithm flow chart

This research integrates multiple machine learning and text processing methods to devise a hybrid method applicable to domains of specific medical literature retrieval. The flow chart of the algorithm is depicted in Fig. 2. A hybrid algorithm consisting of a two-stage information retrieval method based on the improved BM25 algorithm, k-means clustering, and BioBert model to better determine the most relevant biomedical articles to specific diseases, genes, and individual traits.

The improved BM25 algorithm computes three scores for the vocabulary, co-word, and expanded word that lead to a composite retrieval function whose parameters are optimized by the cuckoo optimization algorithm. Afterward, the BioBert pre-trained model is utilized to generate a sentence vector for each sentence in the abstract to obtain a sentence-level vector representation, which is a 1 × 768 dimensional vector. Sentences are then clustered by using the K-means clustering regarding the closest sentence to the center of a cluster of the category until the overall length reaches 512 tokens to form a new abstract text. Finally, the output of the BERT model that is employed in the BioBert-based document similarity matching method is utilized to obtain the similarity between the document and the retrieved morphemes.

To exemplify what has been conducted, first, patient information and medical articles are input into the system, such as patient information, disease, demographics, genes, and other attributes. Medical article information includes title, abstract, MeSH headings, chemical list, and keyword list. The patient information was input into the MeSH library to obtain the expanded query information, and the patient information and the expanded word information were input into the improved BM25 algorithm [13] to obtain the abstract score, word score, and co-word score, which were then standardized and processed according to the standardization process. Afterward, the top 1000 articles were sorted in descending order by using their composite retrieval scores. The abstract and title similarity scores of each document and the query were calculated by using the BioBert document similarity matching method for the top 1000 articles. The standardized scores were then added to the improved BM25 scores, and the final scores were sorted in descending order to reflect the similarity scores.

### Structured data

Table 1 summarizes the evaluation results obtained between 2017 through 2019 for the initial screening of the literature. It is a screening factor for human precision medicine (PM), and the co-occurrence of disease genes is also an important factor for determining the correlation. Therefore, the co-word method proposed in the improved BM25 algorithm [13] can increase the scores in potentially relevant articles. When the search elements are defined, the term “human” as one of the search elements of the baseline is utilized to distinguish between humans and animals. Because the PM tasks in 2020 and between 2017 through 2019 were different, and demographics were replaced by treatment, the tasks in 2020 are excluded and the tasks between 2017 through 2019 are used as PM retrieval tasks for the research data. Table 2 shows the PM retrieval tasks between 2017 through 2019. Observed that disease and genes are fixed expressions, and age and gender need to be classified during retrieval. The classification criteria are shown in Table 3. The regular expression extracts the age from the abstract, such as years-old/year-old/years old, which are all extracted to form the corresponding category, and the word stem of nltk is used to extract the words that express gender in the abstract, such as woman, man, girl, and boy. If the abstract does not contain demographic information, matching items from the Mesh for extraction are searched for.

**Table 1:** Raw judgments for Scientific Abstracts

Pm_rel	Disease	gene1_annotation	gene1_name	gene2_annotation	gene2_name
Human PM	Exact	Missing Gene	NRAS(Q61K)	Exact	TP53
Not PM	Not Disease	Exact	KRAS	Missing Gene	KRAS

**Table 2:** PM tasks between 2017 through 2019

Topic	Disease	Gene	Demographic
2017–1	Liposarcoma	CDK4 Amplification	38-year-old male
2018–2	Melanoma	BRAF (V600E)	64-year-old male
2019–3	Melanoma	BRAF (E586K)	64-year-old female

**Table 3:** Demographic classification

Demographic variables	Values	Categories
Age	Fetus	Fetus
	Birth to 1 month	Newborn
	1 month to 24 month	Infant
	2 years to 6 years	Preschool
	6 years to 13 years	Child
	13 years to 19 years	Adolescent
	19 years to 35 years	Young
	35 years to 60 years	Middle age
	60 years to 80 years	Aged
	Over 80 years	Aged 80
	Over 18 years	Adult
Gender	Female, woman, girl	Female
	Male, man, boy	Male

### Generation of the training sample

Through the analysis of data sets between 2017 and 2019, we divided the search tasks into two types: the same gene with different diseases and the same disease with different genes. While different diseases with the same gene are shown in Table 4, different genes with the same disease are presented in Table 5. To eliminate the interference of the search task and document matching, disease, gene, and demographic information from the head of the abstract are extracted and negative samples for the content of the same disease with different genes or different diseases are generated, as shown in Table 6.

**Table 4:** Different diseases with the same gene

Topic	Disease	Gene
2017–12	Colon cancer	BRAF (V600E)
2018–1	Melanoma	BRAF (V600E)
2017–5	Melanoma	BRAF(V600E), CDKN2A Deletion

**Table 5:** Different genes with the same disease

Topic	Disease	Gene
2018–1	Melanoma	BRAF (V600E)
2018–2	Melanoma	BRAF (V600K)
2018–3	Melanoma	BRAF (V600R)

**Table 6:** Negative samples

PMID	Document	Query	Topic	Label
10,101,594	cdk4 amplification human middle-aged adult male + summary	Liposarcoma cdk4 amplification middle-aged male	2017–1	1
10,101,594	cdk4 amplification human middle-aged adult male + summary	Liposarcoma mdm2 amplification and a young male	2017–20	0

## Experimental results

### Data

The Text Retrieval Conference (TREC) has been launched for biomedical article retrieval tracks for seven consecutive years. TREC 2014–2016 [51–53] focused on the full-text retrieval of biomedical articles, while TREC 2017–2020 [54–57] focused on article retrieval for precision medicine (PM).

The data sources are mainly divided into baseline data and evaluation datasets. The baseline data set uses the PubMed literature metadata download provided by the organizing committee of TREC. The specific data are shown in Table 7. The metadata used includes PMID, titles, abstracts of articles, Chemical words, Mesh words, and keywords.

**Table 7:** Metadata details

Name	Values
Abstract-number	29,137,637
Title-number	29,137,637
Chemical-number	13,670,358
Mesh-number	25,389,659
Keyword-number	5,435,471

In the 2017–2019 TREC-PM tasks, a total of 120 patient cases and 63,387 qrels (document correlation judgment) were available, as shown in Table 8.

**Table 8:** Evaluation datasets

Year	Queries	Documents (rel./irrel.)
2017	30	3,875/18,767
2018	50	5,588/16,841
2019	40	5,544/12,772

### The parameter setting of the proposed algorithm

The adjustment factors of our baseline improved BM25 algorithm [13] use common conventional parameters presented in Table 9. In the document similarity matching algorithm, we performed similarity matching for the abstract and the title, and the query because the lengths of the abstract and the title were significantly different. Therefore, we used different parameters for training, and the settings for the training parameters of the matching degree algorithm are shown in Table 10.

**Table 9:** Parameter settings of the improved baseline BM25 algorithm

Parameters	Value	Remarks
k1	1.2	adjustment factors
k2	1.2	adjustment factors
b1	0.75	adjustment factors
b2	0.75	adjustment factors
avgdwl	85	The average document length (after running word frequency processing)
avgdl	13	The average number of word lists

**Table 10:** Training parameters of the similarity matching algorithm

Type	Parameters	Value	Remarks
Abstract	Epoch	4	Number of training rounds
	Batch_szie	32	Minimum training batch
	Max_len	512	Maximum number of words in a document
	Learning_rate	0.0005	Algorithm learning rate
Title	Epoch	4	Number of training rounds
	Batch_szie	64	Minimum training batch
	Max_len	128	Maximum number of words in a document
	Learning_rate	0.0005	Algorithm learning rate

### Experimental comparison

**Fig. 3:**
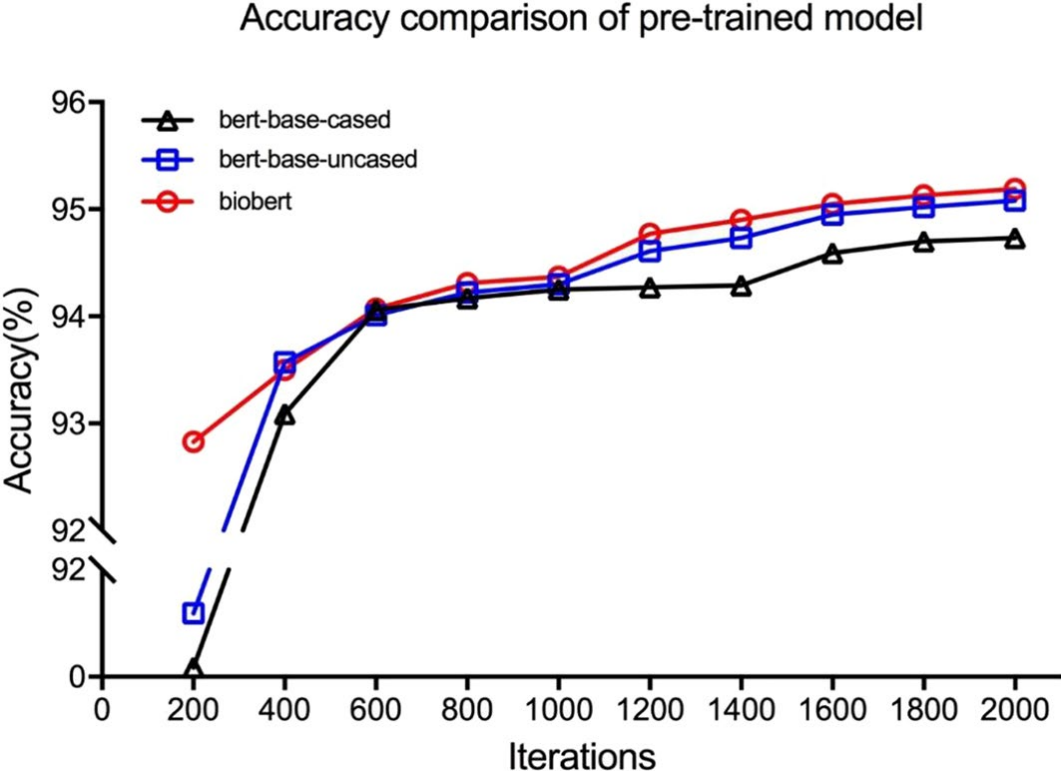
Accuracy comparison of the pre-trained models

Similar to the literature [58], we used the data in 2017 for evaluation and the data in 2018 for training. Besides, while 80% of the data is used for the training phase, 20% of the data is utilized for validation. We used the BioBert model as a pre-training model to generate word vectors, as shown in Table 11. The precision of the proposed method is slightly lower than that of the method proposed in the literature [58], but the recall rate and F1 score of the training set, and the accuracy rate, recall rate, and F1 score of the validation set are found to be higher since the method of negative sample generation is utilized to reduce the interference between similar samples, thus, the official Bert-base-uncased is replaced by the Biobert model. Figure 3 depicts that all 3 algorithms converged at approximately 2000 iterations. When comparisons are conducted, the BioBert converges faster, but its improvement in accuracy is not very significant, which is slightly higher than Bert-base-uncased and Bert-base-cased algorithms.As shown in Table 12, BioBert also has a lower loss rate of 0.11 than that of Bert-base-uncased and Bert-base-cased, which is 0.12.

**Table 11:** Comparison of similarity matching algorithms

Datasets	P	R	F1
Training [19]	0.9814	0.9384	0.9594
Validation [19]	0.9266	0.9147	0.9206
Training	0.9636	0.9656	0.9641
Validation	0.9519	0.9552	0.9530

**Table 12:** The comparison of the pre-trained models

Name	P	Loss
Bert-base-cased	0.9473	0.12
Bert-base-uncased	0.9508	0.12
BioBert	**0.9519**	**0.11**

The significance of bold means optimal values

**Fig. 4:**
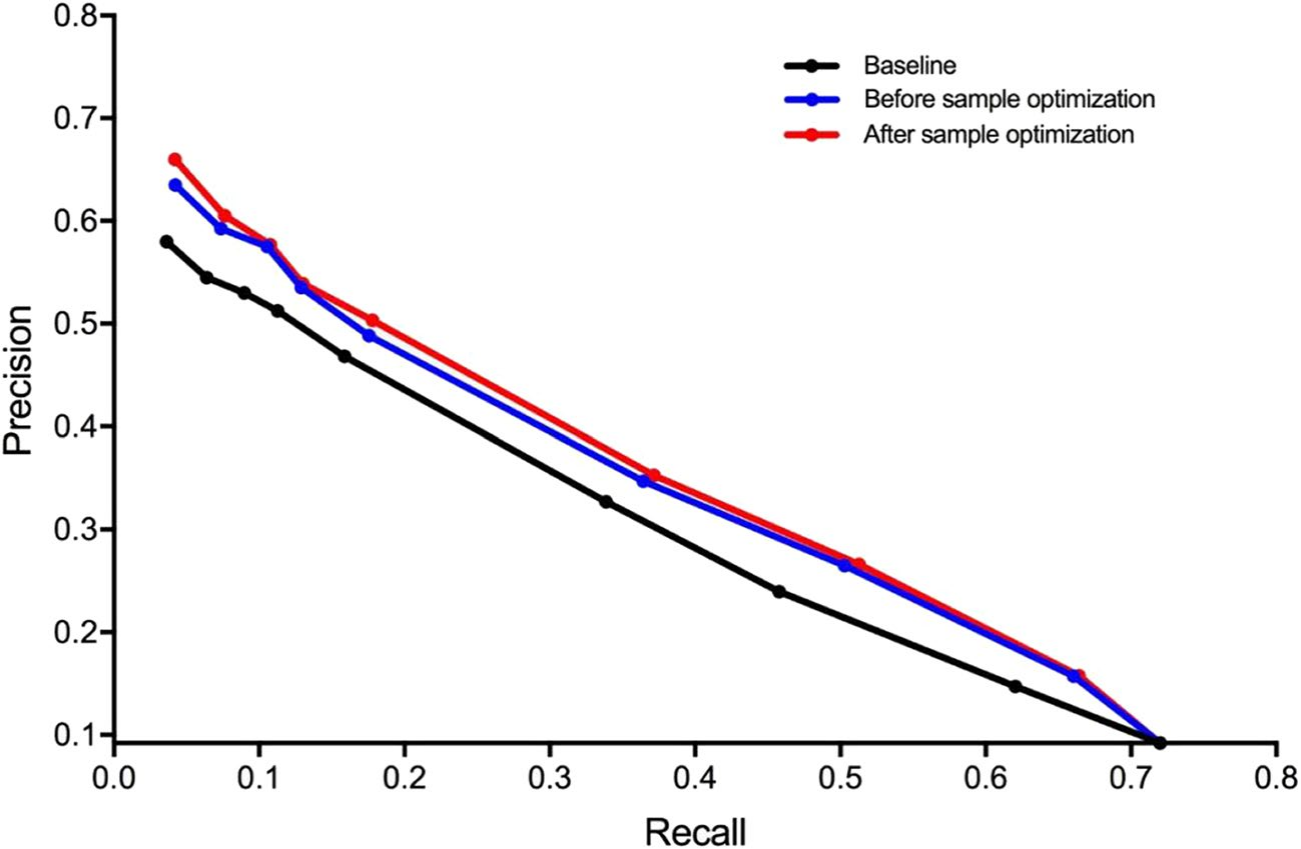
RP curve information system

Table 13 shows the comparison of various indicators of the proposed algorithm before and after the generation of negative samples. The training set with added negative samples has improved outcomes on MAP, NDCG, P@10, and R-Prec, from 0.2928, 0.603, 0.5925, and 0.3503 to 0.3028, 0.6155, 0.6050 and 0.3524, respectively. To verify the improvement of the effect of the negative sample generation method, we used the accuracy and recall rates of 5, 10, 15, 20, 30, 100, 200, 500, and 1000 articles in the top 1000 articles to generate the PR curve, as shown in Fig. 4.

**Table 13:** Comparison of samples before and after optimization

Methods	MAP	NDCG	P@10	R-Prec
Baseline: The improved BM25	0.2663	0.5836	0.5450	0.3138
Baseline (before sample optimization)	0.2928	0.6034	0.5925	0.3503
Baseline (sample optimization)	**0.3028**	**0.6155**	**0.6050**	**0.3524**

The significance of bold means optimal values

The overall curve shows a downward trend with some slight fluctuations. When the PR curve is located above the other PR curves, it means that the performance would reach higher than the other methods. Figure 4 shows that the red curve after sample optimization is located above the curve of the baseline (black) and the one obtained before sample optimization (blue).

**Fig. 5:**
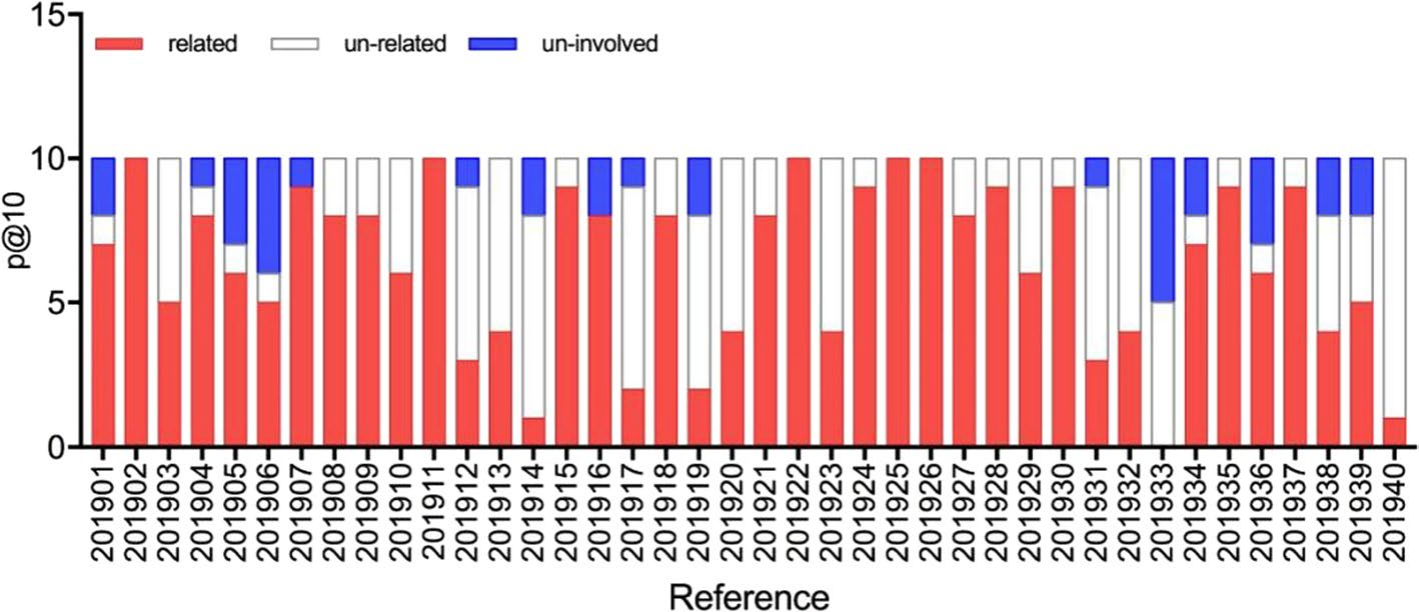
The schematic diagram for the proportions of the three types of P@10 in the literature

Table 14 shows the experimental comparison between the proposed algorithm and the state-of-the-art algorithm selected [59] in the 2019 TREC PM track. Even though the results of the proposed algorithm are lower than those of the algorithms selected in the 2019 TREC meeting, the evaluations were conducted by a software called the trec_eval software. Seen that the proposed algorithm uses the result of the addition of the baseline score and the abstract similarity score, which are 0.635 (P@10) and 0.344 (R-Prec). These two indicators are slightly inferior to the optimal results of the selected algorithm in that year, which is ranked second. However, we found that among the top 10 articles of the 40 topics, 366 documents that existed in qrels and 34 documents that did not exist in qrels were retrieved, as shown in Fig. 5. Namely, all the 34 documents used to calculate P@10 that did not participate in the evaluation are judged irrelevantly. However, the proposed algorithm still achieved a P@10 of 0.635 without it. If these non-participating documents had been removed from the top10, the P@10 and R-Prec scores of the proposed algorithm would reach 0.68 and 0.4823, respectively.

**Table 14:** The comparison of TREC tasks in 2019

Methods	p@10	R-Prec
BITEM PM	0.6275	0.3166
Julie-Mug [59]	**0.6530**	**0.3572**
Baseline + abstract	0.6350	0.3444
Baseline + abstract + title	0.6050	0.3524

The significance of bold means optimal values

Figure 5 shows that topics have more relevant articles, such as topic 1, topic 4, topic 7, and topic 16, the uninvolved articles still have the potential to be identified as relevant articles. If the title similarity scores had been added, P@10 would decrease to 0.605, but the R-Prec would increase to 0.352, which is already very close to the optimal values of the selected method in that year.

**Fig. 6:**
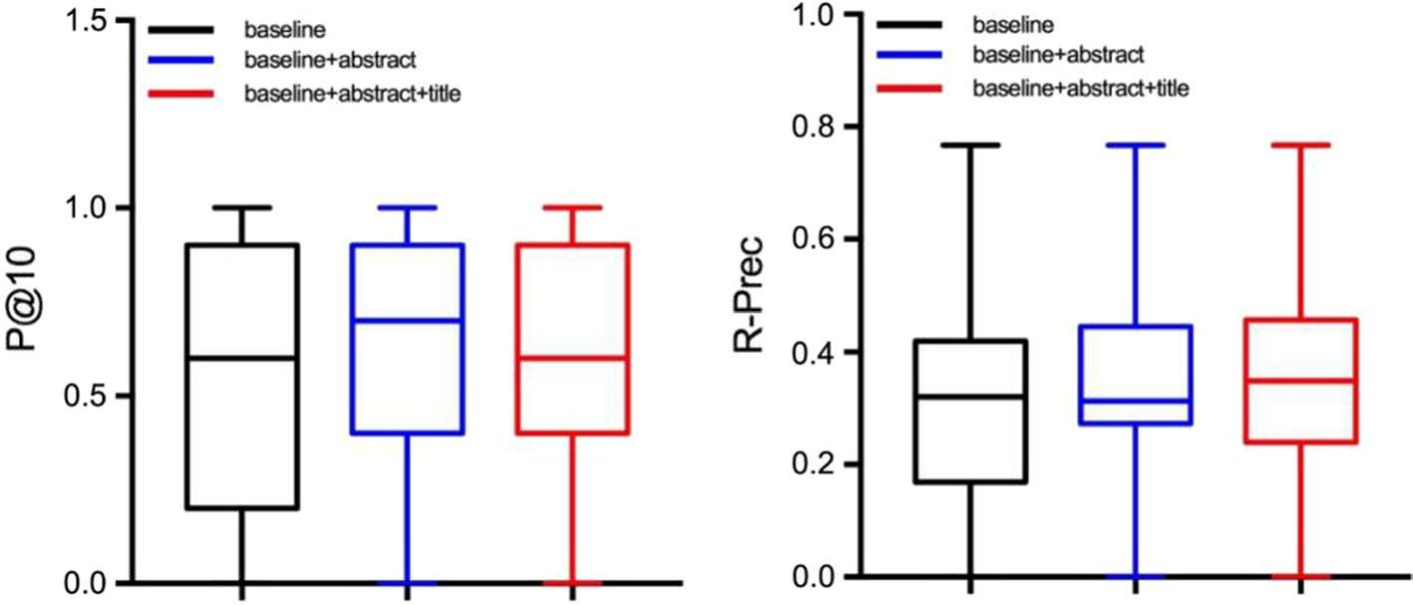
The box-plot representation of P@10 and R-Prec concerning the three algorithms

Figure 6 shows that the addition of the abstract and title scores to the baseline score significantly improves the P@10 and R-Prec of the information system. When P@10 is a concern, the stability of baseline + abstract and baseline + abstract + title is found to be similar. However, there are more uninvolved studies in baseline + abstract + title than in baseline + abstract, which leads to a decrease in P@10. Because the baseline + abstract + title was optimized twice, it was easier to improve the ranking of the potentially relevant literature, but it also increased the ranking of the highly distracting literature, so it looks more polarized than the baseline + abstract.

To further verify the effectiveness of the proposed algorithm, we also select 80% of the data in the 2017–2018 qrels as the training set, 20% of the data as the validation set, and use the PM in 2019 as the task [58]. Just the literature that participated in the evaluation was used as the baseline, and the top 500 retrieved documents were used to submit the evaluation. The experimental comparison results are shown in Table 15. The P@10 and R-Prec of the first search were relatively low at 0.52 and 0.2307, respectively. After using the secondary sorting algorithm, the P@10 and R-Prec were significantly improved, reaching 0.6750 and 0.3912 with Baseline + REL, and Baseline + REL + ABS reached 0.6985 and 0.3627. In contrast, the baseline retrieval algorithm of the proposed algorithm achieves 0.5775 P@10 and R-Prec, respectively in one retrieval. Baseline + Abstract reached 0.6725 and 0.4636, and Baseline + Abstract + title reached 0.6725 and 0.4716, respectively. Seen that the P@10 of the proposed algorithm is slightly lower than that of the algorithm proposed in the literature [58], while the R-Prec is much higher.

**Table 15:** The comparison of the algorithms

Methods	P@10	R-Prec	P@10*R-Prec
Baseline: SolreDisMax	0.5200	0.2307	0.1200
Baseline + REL	0.6750	0.3912	0.2641
Baseline + REL + ABS	**0.6985**	0.3627	0.2533
Baseline: improved BM25	0.5775	0.4225	0.2440
Baseline + abstract	0.6725	0.4636	0.3118
Baseline + abstract + title	0.6725	**0.4716**	**0.3172**

The significance of bold means optimal values

There are two main reasons: (1). The results of the algorithm used in the first round of the search in the literature [58] were not functioning well. (2). The Implementation details were mentioned as follows: [58]: “All parameter choices were made based on the best practices from prior efforts and experiments to optimize P@10 on validation subsets”. Because of the intervention of manual experience and special optimization of the P@10 index, it resulted in a higher P@10. However, optimizing for a certain indicator would reduce the universality of the implemented algorithm.

Therefore, the proposed algorithm has the advantage of not conducting an optimization to increase the P@10 index and does not carry out any manual intervention or specified optimization scheme to the indexes, and uses conventional parameters directly. Therefore, the proposed algorithm has a stronger universality than the selected method [58]. Table 15 shows that the optimization of P@10 will produce a certain decrease in R-Prec. Therefore, to comprehensively evaluate the quality of the proposed algorithm, we refer to the calculation method of the F1 score and add an evaluation index represented by P@10*R-Prec. The optimal P@10*R-Prec of the proposed algorithm is found to be 0.3172, while that in the literature [58] is 0.2533, so the proposed algorithm has advantages in terms of universality and comprehensive performance.

## Summary and future work

The manuscript proposes a hybrid algorithm consisting of a two-stage information retrieval method based on the improved BM25 algorithm, k-means clustering, and BioBert model to better determine the most relevant biomedical articles to specific diseases, genes, and individual traits.

The improved BM25 algorithm computes three scores for the vocabulary, co-word, and expanded word that leads to a composite retrieval function whose parameters are optimized by the cuckoo optimization algorithm that retrieved better search outcomes. Afterward, the BioBert pretraining model is utilized to generate a sentence vector for each sentence in the abstract to obtain a sentence-level vector representation, which is a 1 × 768 dimensional vector. Sentences are then clustered by using the K-means clustering regarding the closest sentence to the center of each category until the overall length reaches 512 tokens to form a new abstract text. Finally, the BioBert-based document similarity matching method is utilized to obtain the similarity between the document and the retrieved morphemes. Besides, negative sampling for the training data is implemented to enhance the accuracy of the proposed method.

The proposed algorithm does not carry out any manual intervention or special optimization schemes to increase the index scores and uses conventional parameters to attain better search or text-ranking outcomes, which guarantees the universality of the proposed algorithm.

To verify the effectiveness of the proposed algorithm, a comparison study is conducted with the state-of-the-art algorithm [58], the proposed algorithm has advantages in terms of universality and better measurement scores. The comprehensive performance analysis of the proposed algorithm shows that a 3% increase of P@10 than that of the state-of-the-art algorithm in TREC 2019 is achieved. Moreover, to comprehensively evaluate the quality of the proposed algorithm, we refer to the calculation method of the F1 score and add an evaluation index represented by P@10*R-Prec. The optimal P@10*R-Prec of the proposed algorithm is found to be 0.3172, while that in the literature [58] is found to be 0.2533.

Consequently, the proposed algorithm has advantages in terms of universality and comprehensive performance.

In future work, the tasks that were negatively affected by the proposed algorithm are analyzed to improve its performance. Besides, different combinations of algorithms dealing with different retrieval scenarios are investigated to thus improve retrieval accuracy.

## Data Availability

The data used in this work were sourced from medical articles published in 2017, 2018, and 2019 TREC Precision Medicine, which can be found at http://www.trec-cds.org/2017.html, and http://www.trec-cds.org/2019.html, respectively. Each article was formatted using XML 2017. The assessment results of the articles were obtained from https://trec.nist.gov/data/precmed/qrels-fnal-abstracts.txt, https://trec.nist.gov/ data/precmed/qrels-treceval-abstracts-2018-v2.txt, and https://trec.nist.gov/ data/precmed/qrels-treceval-abstracts.2019.txt.

## Abbreviations

TF-IDF: Term frequency-inverse document frequency
BM25: Best matching 25
IR: Information retrieval
TREC: The text retrieval conference
BERT: Bidirectional encoder representation from transformers
